# Social Support and Emotional Intelligence as Protective Resources for Well-Being in Moroccan Adolescents

**DOI:** 10.3389/fpsyg.2019.01529

**Published:** 2019-07-10

**Authors:** Esther Lopez-Zafra, Manuel Miguel Ramos-Álvarez, Karima El Ghoudani, Octavio Luque-Reca, José María Augusto-Landa, Benaissa Zarhbouch, Smail Alaoui, Daniel Cortés-Denia, Manuel Pulido-Martos

**Affiliations:** ^1^Department of Psychology, Social Psychology, University of Jaén, Jaén, Spain; ^2^Department of Psychology, Methodology of Behavioral Sciences, University of Jaén, Jaén, Spain; ^3^Faculty of Education and Psychology, Francisco de Vitoria University, Pozuelo de Alarcón, Spain; ^4^Department of Psychology, Faculty of Human Sciences – Dhar El Mahraz, Sidi Mohamed Ben Abdellah University, Fez, Morocco

**Keywords:** adolescents, emotional intelligence, life satisfaction, social support, depression

## Abstract

This study aimed to test a structural model to examine the protective role of psychosocial variables, such as social support, emotional intelligence and their interaction, on the cognitive dimension of subjective positive well-being (life satisfaction) and negative well-being (depression) in Moroccan adolescents. The participants consisted of 1277 students (571 men, 694 women and 12 missing values) with a mean age of 16.15 years (*SD* = 2.22; range = 9 to 23) who attended 26 public schools in different territories of Morocco. These students were in secondary education (*n* = 893) and high school (*n* = 378) (6 missing values). The scales for measuring the variables of interest had to be adapted and validated as a previous step for the further proposal of a model of relations. Statistical analyses were conducted using structural equation modeling (SEM) to test the proposed model. The model that optimally adjusted the data confirmed the protective role of social support in the well-being of Moroccan adolescents. Consistent with previous studies, social support was directly related to well-being. However, it also modulated levels of satisfaction with life. Likewise, the inclusion of emotional intelligence as an additional protective factor contributed to the explanation of the well-being mechanisms in adolescents. In addition to direct associations with the levels of social support, satisfaction with life and depression (negative in the latter case), emotional intelligence participated in a complex chain affecting life satisfaction and life satisfaction affecting depression. Moreover, the interaction of emotional intelligence with social support was confirmed to determine levels of life satisfaction in adolescents. Specifically, social support multiplied the effects of the relationship between satisfaction with life and emotional intelligence in cases of moderate and high levels in Moroccan adolescents. This study fills a gap in the literature by adapting and further analyzing several scales with Moroccan samples of adolescents and by proposing and verifying a relational model that can help researchers and teachers to more precisely clarify these relations according to their context. The enhancement of protective factors, such as social support and emotional intelligence, will promote healthy youth development, thus creating healthier societies in the future.

## Introduction

Adolescence is generally considered a dynamic and complex age period. Furthermore, there are differences among countries depending on culture (Arnett, 2018), and thus, the results of studies about this period of life may not be generalized to all cultures. There is a wide body of research about adolescence and its implications for psychological boundaries in the United States and Europe (Jackson and Goossens, 2006), along with the relations among well-known psychological constructs in Western countries (such as emotional intelligence or well-being), whereas there is a dearth of studies in other cultures, such as those of northern Africa. In Morocco, adolescence (*sinn al-murahaqa*) is not a widely recognized stage of life. Only in recent decades is it usually associated with the years that coincide with high school education, and it is distinguished by the development of *aql*, a term referring to social responsibility (Davis and Davis, 2012). Typical behaviors of this stage begin with puberty in the mid-teens for both sexes (beginning earlier in girls than in boys) and extend until marriage, coinciding with the years of training in and learning adult roles; these years are also marked by a lack of economic independence (Gregg, 2005). In this period of life, youth development includes feelings of positivity or negativity and consideration of the consequences of one’s actions in adulthood. Their satisfaction with life as a positive aspect of well-being or the absence of depression as a suppression of negative well-being could be affected by other variables. In general, the role of social support is accepted as a protective factor for well-being, but we propose the exploration of the protective role of emotional intelligence to further contribute to knowledge about their relations in Moroccan adolescents.

Definitions of subjective well-being distinguish an affective and a cognitive component (Diener et al., 1999). The affective component is an individual’s (actual or perceived) hedonic balance (i.e., the balance between pleasant affect and unpleasant affect). The cognitive component is an individual’s life satisfaction (i.e., global evaluations of one’s life according to subjectively determined standards) (Pavot and Diener, 2008) and is related to health predictors such as favorable self-reported health, social support and positive health behaviors (Koivumaa-Honkanen et al., 2004). However, other empirical studies suggest that positive and negative components of well-being are influenced by different factors that are not simply the opposite ends of a continuum (Chamberlain, 1988). In fact, we posit that it is necessary to study both positive and negative components of well-being and the variables that could affect them. For example, life satisfaction as a positive component of well-being can be determined by diverse factors, such as self-esteem, income, social support, individualism versus collectivism, and cultural homogeneity, among others (Diener and Diener, 1995). Thus, social support could predict life satisfaction, but we could also wonder about their relationship in adolescence. Studies on adolescence highlight that life satisfaction constitutes a relevant predictor of psychological adjustment variables such as depression (Evans et al., 2005; Proctor et al., 2009). Thus, previous empirical evidence suggests that having high levels of life satisfaction protects adolescents against the development of psychological disorders (Proctor et al., 2009). One of the disorders that has increased dramatically during adolescence and poses potential danger (i.e., from affecting academic performance to influencing decisions to commit suicide) is depression (Brière et al., 2013). According to the World Health Organization (WHO), depression is a medical disease related to negative psychosocial outcomes, characterized mainly by symptoms such as sadness, loss of interest or inability to experience pleasure, feelings of guilt and low personal worth, appetite and/or sleep disturbances or concentration problems (World Health Organization, 2017a). Depressive symptomatology constitutes one of the main threats to which teens around the world are exposed (World Health Organization, 2017b).

In the case of Arab adolescents, the Institute for Health Metrics and Evaluation (IHME) estimates that approximately 25% of cases of disability and premature death are due to mental and behavioral disorders, and depression ranks first (Institute for Health Metrics and Evaluation [IHME], 2013). In addition, depression is expected to become the main mental health problem among adolescents in Arab countries (Institute for Health Metrics and Evaluation [IHME], 2013). In fact, studies conducted in countries such as Jordan or Saudi Arabia find a prevalence of depression in adolescents between 30 and 38% (Al-Gelban, 2007; Raheel, 2015; Dardas et al., 2018). Although it is fundamental to explore risk factors that increase the probability of Arab adolescents experiencing depressive symptomatology (Dardas et al., 2018) and the cognitive variables that are responsible for the increase in depression experienced during the adolescence stage (Abela and Hankin, 2008), the tradition of investigating protective factors that attenuate or eliminate the influence of risk factors on depression is less developed (i.e., resilience, Carbonell et al., 2002 or life satisfaction, Lyons et al., 2014). There are several studies confirming an association between life satisfaction and depression in adolescents in Western countries (Gilman and Huebner, 2006) and among Arab teenagers (Abdel-Khalek, 2009; Abdel-Khalek and Eid, 2011). To further understand this relationship in depth, it is essential to clarify its directionality, both in young and adult populations (Lyons et al., 2014). In this sense, longitudinal studies have confirmed the predictive role of life satisfaction in depression in adult samples (Koivumaa-Honkanen et al., 2004; Boyraz et al., 2014) and adolescents (Huebner et al., 2000; Haranin et al., 2007; Lyons et al., 2014). Based on these aspects, we propose the following:

Hypothesis 1. Life satisfaction is negatively related to depression in Moroccan adolescents.

Social support has long been known to exert considerable influence on wellbeing (Thoits, 2011). Social support is defined, in a broad sense, as the set of human and material resources available to an individual to help them overcome a certain crisis situation (Lin, 1986) and cope with stress (Cohen, 2004). These resources can be real or only perceived and are based on two types of social support: structural social support, which has to do with the closest quantitative resources (i.e., family and friends), and qualitative social support, whose purpose is to help the individual in their performance (i.e., work team, teachers, and counselors) (Cobb, 1976; Gottlieb, 1983).

Social support is hypothesized to protect well-being both directly, through the benefits of social relationships, and indirectly, as a buffer against stressful circumstances (Gariépy et al., 2016). The *direct approach* posits that positive perceptions of social support have a direct positive effect on health and well-being, regardless of stress (Berkman et al., 2000). Thus, the mere fact of being in a supportive social network of family and friends could directly improve general health and well-being (Uchino, 2004), contributing to life satisfaction, mainly the support received by family and friends (Castellá Sarriera et al., 2015). Furthermore, support from parents and classmates increases adolescents’ well-being and school environment (Rueger et al., 2010).

However, despite the well-established importance of these sources of support for adolescents (Criss et al., 2009), they may also function in a different way. In fact, two meta-analyses aiming to shed light on this point found different results. Rueger et al. (2016) found that family and general peer support emerged as the strongest sources of support, followed by teacher and close friend support, whereas Chu et al. (2010) found that teacher support emerged as the strongest correlate of well-being, followed by family and peer support. Thus, regardless of the source of social support, it is important to perceive social support to have positive life satisfaction, and thus, the following hypothesis is proposed:

Hypothesis 2. Perceived social support is positively related to life satisfaction in Moroccan adolescents.

In the general population, the systematic review by Santini et al. (2015) provides some confirmation that perceived social support plays important protective roles against depression. Additionally, the systematic review by Gariépy et al. (2016) found that parents, teachers and family were sources of support who were most consistently found to be protective against depression in children and adolescents, whereas findings were less consistent for support from friends and general perceived support. Moreover, in adolescents, parental support is important to buffer the positive association between depression and adolescents’ reports of suicidal ideation (Fredrick et al., 2018). In fact, the association between depression and suicidal ideation was not significant for students with high parental support, regardless of gender. In sum, support from parents and family is, more than any other source, most consistently related to a youth’s protection from depression (Gariépy et al., 2016), proving to be effective in the mitigation of stress perceptions and depressive symptomology (Crutcher et al., 2018). Thus, we consider the following:

Hypothesis 3. Perceived social support is negatively related to depression in Moroccan adolescents.

In recent years, the emergence of positive psychology has promoted the study of emotional abilities that could impact life satisfaction or depression, such as emotional intelligence. Under the ability model paradigm, emotional intelligence comprises the individual differences that occur when meaning is given to the emotional patterns present in people’s lives and how those patterns are used to reason and solve problems (Salovey and Mayer, 1990; Mayer and Salovey, 1997). It covers the abilities to perceive and express emotions, to take into account the emotions when thinking or making decisions, to understand and identify emotions and to regulate emotions in oneself and in others (Mayer and Salovey, 1997; Mayer et al., 2000). Although the personality and dispositional attributes targeted by the mixed/trait models also contribute to reasoning and problem solving, they should not be confused with emotional intelligence as a mental ability that is discrete and measurable (Mayer et al., 2008a).

Emotional intelligence plays a key role at the adaptive level, and its usefulness has been demonstrated in different contexts (social, academic, and labor) (Mayer et al., 2008b; Zeidner et al., 2008), showing solid relationships with levels of well-being (Zeidner et al., 2012). In the meta-analysis conducted by Sánchez-Álvarez et al. (2016) regarding the contribution of emotional intelligence to subjective well-being, the authors conclude that emotional intelligence is consistently and positively related to subjective well-being, and the effect sizes are higher when emotional intelligence is evaluated through self-report measures; this result coincides with those of previous review works, such as Martins et al. (2010). Moreover, the study by Sánchez-Álvarez et al. (2016) highlights a higher effect of emotional intelligence on the cognitive than on the affective dimension of well-being (Palmer et al., 2002), which could be justified by the higher temporal stability of both variables (emotional intelligence and the subjective well-being cognitive dimension) vs. the affective component (Sánchez-Álvarez et al., 2016), along with the implication of meta-cognition processes that would share the evaluations of emotional intelligence and cognitive judgments on the general subjective level (Mayer and Stevens, 1994). Thus, we propose the following:

Hypothesis 4. Emotional intelligence is positively related to life satisfaction (cognitive dimension of subjective well-being) in Moroccan adolescents.

Emotional intelligence also preserves the well-being of adolescents due to its relationships with depressive symptomatology (Balluerka et al., 2013; Resurrección et al., 2014). One explanation is that emotional intelligence reduce the experimentation and duration of negative emotions (Mikolajczak et al., 2009a; Zeidner et al., 2009), and thus, these emotional abilities act as protective factors against the development of psychological maladjustment (Mayer and Salovey, 1997), enabling effective selection of coping strategies and conditioning their effectiveness (Davis and Humphrey, 2012). Thus, we propose the following:

Hypothesis 5. Emotional intelligence is negatively related to depression in Moroccan adolescents.

As previously mentioned, social support can provide a source of buffering of the negative effects that adverse conditions have on well-being. They also enhance the development of adolescents’ well-being. In fact, young people at these ages depend to a large extent on different forms of social support and how they affect different spheres of life (Stanton-Salazar and Spina, 2005). For example, promoting adaptive career development by increasing their confidence in their emotional skills, which would impact their expectation of social support (Fabio and Kenny, 2012). Thus, it would be expected that emotional intelligence would allow young people to establish and maintain closer social relationships as well as obtain higher levels of social support (Saarni, 1999). In fact, studies using different procedures of evaluating emotional abilities have found similar results; that is, emotional intelligence is related to social support (Ciarrochi et al., 2001, 2002b) and to the quality of interpersonal relationships (Lopes et al., 2003). However, there is a lack of studies in Moroccan samples. Thus, we propose the following:

Hypothesis 6. Emotional intelligence is positively related to perceived social support in Moroccan adolescents.

Even when emotional intelligence consistently shows relationships with subjective well-being (Sánchez-Álvarez et al., 2016) and levels of depression in adolescents (Balluerka et al., 2013; Resurrección et al., 2014), there is a lack of knowledge about the mechanisms through which these relationships are established. The consideration of emotional intelligence as a variable that interacts with the perception of environmental elements in the explanation of health levels is a constant in research (Ciarrochi et al., 2002a; Slaski and Cartwright, 2003). For example, in work contexts, it has shown an invigorating role that allows for a better interpretation and understanding of the social keys and more positive and adaptive responses (Jordan et al., 2002). In areas other than work, Gallagher and Vella-Brodrick (2008) demonstrated in a sample of adults from the general population that emotional intelligence interacted with the levels of received social support by increasing the resulting levels of subjective well-being. However, in adolescents who are in an academic context, there are other variables that may affect the relationship between social support and well-being, for example, health variables such as social stress, anxiety or depression (Demaray and Malecki, 2002). In this sense, the second approach has focused on the *stress-buffering* hypotheses (Vaux, 1988), which posits that social support suppresses the deleterious effects of stress to promote or maintain good health (Glozah, 2013). The results under this paradigm show, in children and adolescents, that social support is a buffer for stress and positively correlates with well-being (Malecki and Demaray, 2006). Furthermore, social support both directly and indirectly affects depressive symptoms and significantly mediates the effects of undesirable life events (Lin et al., 1999). Bearing these comments in mind and the above-mentioned result about the dramatically increased depression in adolescents that has affected a wide range of college students (Hunt and Eisenberg, 2010), we consider the possible protective effect of social support on depression. Stemming from these results, we could expect that Moroccan adolescents with high levels of emotional intelligence place greater value on the interaction experiences resulting from the social support received from different sources than do adolescents with lower emotional intelligence levels, further leading to greater life satisfaction (cognitive dimension of subjective well-being) and lower levels of depression. Thus, emotional intelligence would have an interaction role with social support in the final result of life satisfaction and depression. Specifically, we propose:

Hypothesis 7. Emotional intelligence interacts with social support in determining levels of life satisfaction in Moroccan adolescents.

Hypothesis 8. Emotional intelligence interacts with social support in determining levels of depression in Moroccan adolescents.

**FIGURE 1:**
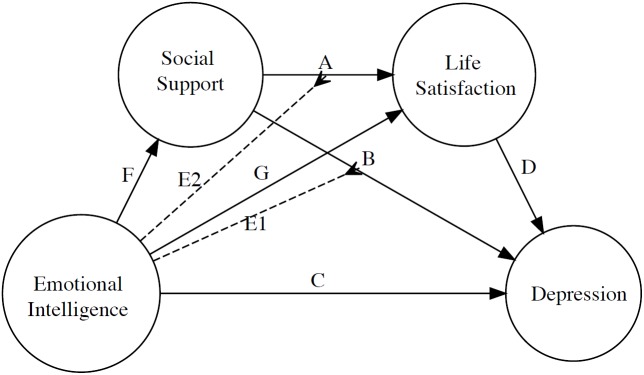
Hypothesized Model.

In sum, our study pays attention to the protective role that social support, emotional intelligence and their interaction may have in life satisfaction and depression in Moroccan adolescents. This is a novel approach to a sample that has received little attention. The hypothesized model is schematized in Figure 1.

## Materials and Methods

### Participants and Procedure

The participants in this study consisted of 1277 students (571 men and 694 women, and 12 missing values) with a mean age = 16.15 (*SD* = 2.22; range = 9–23 years old) who belonged to 26 public schools in different territories of Morocco. The sample comprised 69.9% secondary education students (*n* = 893; 15.3% first course; 14.7% second course; 19.7% third course; and 20.2% fourth course) and 29.6% high school students (*n* = 378; 7.8% first course and 21.8% second course). The remaining 0.5% (*n* = 6) was missing values. The mean grade of the students in the previous year was 12.84 (over 20; *SD* = 2.56; range = 5.00–19.99). In the family context, the students had more than four siblings (18.1%), four siblings (19.3%), three siblings (23.3%), two siblings (24.7%), one sibling (12.6%), and no siblings (1%). The remaining 1.2% was missing values.

The mean age of fathers was 49.75 years (*SD* = 8.10; range = 30–89), whereas mothers’ mean age was 42.07 years (*SD* = 6.99; range = 26–84). With respect to the parents’ educational level, 20% of fathers had no education, whereas 22.4% completed primary studies, 15.4% graduated from secondary education, 20.3% graduated from high school, and 18.7% had university studies. The remaining 3.1% were missing values. For mothers, 36.6% had no education, and the distribution of mothers in the remaining educational levels was as follows: primary studies (17.8%); graduated from secondary education (16.4%); graduated from high school (14.3%); and completed university studies (12.6%). The remaining 2.3% were missing values. With respect to the parents’ work, fathers were mostly self-employed (32.7%), worked as civil servants (22.5%), or were employees (12.2%), followed by working as farmers (7.3%), being unemployed (6.3%) or holding other unspecified jobs (15.5%), and 3.5% of missing values. Mothers were mostly unemployed but devoted to their houses (72.7%), followed by working as civil servants (8.2%), being self-employed (7%), being employed (4.3%), working as farmers (0.7%), or having unspecified work (5.4%). The remaining 1.7% was missing values.

As a previous step before administering the questionnaires, two processes were carried out. First, once the scales that conformed to the battery were decided, then based on the guidelines by Hambleton and De Jong (2003), all of the scales were revised by experts in the Arabic language and in psychology and were further analyzed in group discussions to test their adequacy among Moroccan adolescents, as suggested by Willgerodt (2003) or Vogt et al. (2004), among others. Due to the content analyses and considering the difficulties and limitations found in other studies with Moroccan samples (i.e., El Rhazi et al., 2009 or Hoopman et al., 2009), several modifications were included. Then, a pilot analysis with Moroccan adolescents was carried out, and other modifications were considered. First, all the scales reduced their number of anchors in the Likert scale. This decision pursued to eliminate the bias of the central response tendency or acquiescence. Thus, the instrument to assess social support was reduced to 2 points (*yes* or *no*), and the instruments for emotional intelligence and life satisfaction were reduced to 4- and 6-point Likert scales, respectively, from *totally disagree* to *totally agree*. Furthermore, several items were linguistically adapted to a simpler form of standard Arabic in all scales, except for the instrument evaluating satisfaction with life, which needed no further changes.

Ethical permission was obtained from the Department of Psychology and was approved by the Research and Ethics Committee at the Faculty of Letters and Human Sciences-Dhar el Mehraz of the University of Sidi Mohamed Ben Abdellah in Fez (Morocco). Once the investigators were allowed to conduct the study, they applied for permission from the responsible parties of the Regional Academy of Education and Training to allow them access to the public schools. The administrative and education officials approved the questionnaire and procedure to be administered at the public schools and gave the investigators a letter to present at the schools. At each school, an internal committee composed of the school personnel, informed the families in a meeting by a written letter with the explanation of the study to obtain parental consent for all participants. Parents verbally consented when they agreed, in case they did not, they had to give the letter back with the petition to be excluded. All parents agreed to allow their children to participate and schools reported the researches with this information. The Ethics Committee approved all consent procedures and how to obtain the parental consent.

In total, twenty-six schools from the region participated in the study. A group of 26 collaborators (24 women and two men) were distributed into two groups (14 and 12 participants, respectively) to be instructed about the scales, the meaning of items and the procedure to administer the questionnaires. They were also instructed to follow the ethical procedure guidelines approved by the Ethic Committee and the Regional Academy of Education and Training. Each seminar lasted 2 h. Then, the collaborators went to the schools in two sessions to have all the scales completed during school hours. Pupils answered the questionnaires individually in the classroom. Students in Morocco are not accustomed to completing questionnaires, and collaborators had to read the questions and sometimes explain the items. Anonymity of the responses and voluntary participation were ensured.

### Measures

*Multidimensional Scale of Perceived Social Support-Arab American* (MSPSS-AA). This is the adaptation of the Multidimensional Scale of Perceived Social Support (MSPSS; Zimet et al., 1988) to Arabic for Arab-American adolescent samples (Ramaswamy et al., 2009). This adaptation included some modifications to culturally adapt the scale: (a) the reduction of the Likert scale from 7 to 3 points; (b) the inclusion of school personnel as a source of social support; and (c) the omission of other significant people as a source of social support due to their romantic nature. As a result, the MSPSS-AA is composed of 12 items comprising three sources of support, namely, family, friends and school personnel, with three response options: *in disagreement, neutral*, and *in agreement*. The MSPSS-AA obtained adequate reliability indices for the three subscales: family (α = 0.63; i.e., my family is close to me when I need them), friends (α = 0.75; i.e., my friends try to help me), and school personnel (α = 0.72; i.e., I talk with the school counselor about my problems) (Ramaswamy et al., 2009). Further modifications were included in our study (see procedure above).

*Wong and Law Emotional Intelligence Scale* (WLEIS; Wong and Law, 2002). The WLEIS is considered a short instrument comprising 16 items that are scored on a 7-point Likert scale and that measure four competencies with four items each: self-emotional appraisal (SEA), which refers to the perception of own emotions; others’ emotional appraisal (OEA), which refers to the perception of the emotions of others; use of emotions (UOE), which refers to the ability of individuals to make use of their emotions by directing them toward constructive activities and personal performance; and regulation of emotions (ROE), the ability of people to regulate their emotions, which will enable a more rapid recovery from psychological distress. The internal consistencies of the competencies in the original version are 0.87, 0.90, 0.84, and 0.83 Cronbach values, respectively (Wong and Law, 2002). The authors initially proposed this instrument for the study of emotional intelligence in the workplace. However, its use has spread to other contexts in which relationships occur (Lopez-Zafra and Gartzia, 2014). For example, it has been successfully adapted and used with Moroccan women by El Ghoudani et al. (2018). We use this adaptation to analyze its usefulness with Moroccan adolescents but further revise it for use among adolescents (see procedure above).

*Satisfaction with Life Scale* (SWLS; Diener et al., 1985). We revised the Arabic version of the SWLS by Ayyash-Abdo and Sánchez-Ruiz (2012) used with a sample of Lebanese undergrads. This scale comprised 5 items rated on a 7-point Likert scale ranging from 1 (*strongly disagree*) to 7 (*strongly agree*), with higher values representing greater satisfaction. One example item is the following: In most ways, my life is close to my ideal. This measure is considered suitable for research and clinical purposes in Arabic-speaking communities showing an adequate alpha coefficient (α = 0.79) in the adaptation study. Our Moroccan version included some adaptations (see procedure above).

*Beck Depression Inventory-II* (BDI-II; Beck et al., 1996). The BDI-II is a 21-item inventory that assesses depressive symptomatology. Each item is rated on a scale ranging from 0 (*normal*) to 3 (*most severe*), with summary scores ranging between 0 and 63. The BDI-II yielded high internal consistency (α = 0.93 among college students; Beck et al., 1996). Adequate content and factorial validity have been demonstrated, and it provides diagnostic discrimination. For this study, we use the version for Arab samples by Hamdi (2013). This scale has been used with the adult population; thus, we reviewed the scale for Moroccan adolescents (see procedure above).

### Data Analysis

Statistical analyses were conducted using structural equation modeling (SEM) to test the proposed model. All were performed using GNU R software (R Foundation for Statistical Computing, 2019) with lavaan, semTools, semPlot, data.table, shiny, HH, irr, vegan, psychometric, psych, GPArotation, Rcsdp, corrplot, parallel, crayon, non-nest2, ltm, CMC, matrixStats, and RMediation libraries. In all cases, a significant difference was determined with a probability = 0.05.

Given the presence of missing values, and in order to avoid interpretive problems related to the normality of the data, the estimation method was based on the *MLF* estimator (Maximum Likelihood Estimation based on the first-order derivatives) and *ml* missing methods (case-wise Maximum Likelihood estimation). In fact, all the conclusions were verified in a convergent manner with other robust estimation methods (e.g., maximum likelihood estimation asymptotically equal to the robust Yuan-Bentler test statistic) (see Rosseel, 2012).

For each model estimated, we report the following recommended goodness-of-fit indices: model chi-square (χ^2^) with degrees of freedom (df) and probability; the ratio χ^2^ value by its degrees of freedom; Hoelter’s Critical N (CN); the Bentler comparative fit index (CFI); Bollen’s incremental fit index (IFI); the non-normed fit index (NNFI, also known as the Tucker-Lewis index, or TLI); the standardized root mean square residual (SRMR); the Steiger-Lind root mean square error of approximation (RMSEA), and Confidence Interval of RMSEA; Akaike’s information criterion (AIC); the sample-size-adjusted Bayesian information criterion (BIC); and the most recent indexes, the gamma hat index (γ-Hat) and the adjusted gamma hat index (adj γ-Hat), which have been shown to be resistant to sample size, model complexity, and model misspecification (Fan and Sivo, 2007). A reasonable guideline is to examine the RMSEA (see below) for the null model (null or independence, e.g., all the variables in the model to have variation but no correlation) and make sure that is not smaller than 0.158, which is especially useful for high sample sizes. This rule allows for the decision of whether incremental (or relative) measures of fit (e.g., IFI, TLI, and NFI) may be informative (Kenny, 2015). The details on the recommended thresholds for each of the goodness-of-fit statistics are included in the Tables 3, 4.

Furthermore, modification indices analysis and their power approach for model fit evaluation were run through the library miPowerFit to debug the final model. This library starts from the estimates of modification indexes of the library lavaan and allows us to include two approaches in a convergent way, namely, that of Saris et al. (2009) from power changes and that based on the confidence intervals of the expected parameter changes (Kelley and Pornprasertmanit, 2016). In the analysis of the moderating effects, we opted for probing latent interaction from products of indicators using residual centering (see Geldhof et al., 2013). In the mediational analysis, the bias-corrected bootstrap method was used to detect mediated effects in SEM to avoid the problems arising from the assumptions about the distribution of the coefficient of interest (Valente et al., 2015).

## Results

### Descriptive Statistics

Means, standard deviations and reliability coefficients for the psychometric measures are presented in Table 1. Pearson product correlations among variables included in the current study are presented in Table 2.

**Table 1:** Descriptive statistics and reliability for all the instruments (*N* = 1277).

Scale	*M* (*SD*)	Range	%NA	Cronbach’s α	OmegaT	OmegaH
MSPSS.F1 Family	7.16 (1.05)	4–8	2.3	0.61	0.62	0.62
MSPSS.F2 Friends	3.54 (0.68)	2–4	1.6	0.48	0.58	0.58
MSPSS.F3 School	5.66 (1.28)	4–8	3.7	0.61	0.62	0.62
MSPSS.TT	16.35 (2.03)	10–20	5.6	0.61	0.69	0.68
WLEIS.F1 Self	9.53 (1.79)	3–12	2.7	0.60	0.62	0.62
WLEIS.F2 Others	12.26 (2.41)	4–16	1.8	0.67	0.68	0.68
WLEIS.F3 Regulation	13.34 (2.20)	4–16	3.2	0.68	0.68	0.69
WLEIS.F4 Use	11.52 (2.91)	4–16	1.2	0.71	0.72	0.71
WLEIS.TT	46.64 (6.57)	18–60	6.8	0.79	0.84	0.83
BDI.F1 Cognitive	25.04 (7.52)	15–57	13.4	0.87	0.88	0.88
BDI.F2 Somatic	7.98 (2.68)	5–20	6.3	0.69	0.67	0.65
BDI.Total	32.85 (9.42)	20–77	15.8	0.89	0.90	0.90
SWLS	21.62 (5.36)	5–30	2.7	0.80	0.80	0.80


*MSPSS, *Multidimensional Scale of Perceived Social Support*; WLEIS, *Wong and Law Emotional Intelligence Scale*; BDI, *Beck Depression Inventory-II*; SWLS, *Satisfaction With Life Scale*; F, name of the factor; TT, total score; NA, no answer/lost values. The α-values < 0.7 obtained for some scales are considered acceptable in the initial phases of research and the development of evaluation instruments (Guilford, 1954).*

**Table 2:** Correlations among dimensions/items.

Dimensions/items	1	2	3	4	5	6	7	8	9	10	11	12	13	14	15	16	17
(1) MSPSS.F1 Family	1																
(2) MSPSS.F2 Friends	0.09^**^	1															
(3) MSPSS.F3 School	0.22^**^	0.06^*^	1														
(4) MSPSS.TT	0.70^**^	0.42^**^	0.77^**^	1													
(5) WLEIS.F1 Self	0.25^**^	0.09^**^	0.17^**^	0.27^**^	1												
(6) WLEIS.F2 Others	0.00	0.13^**^	0.08^**^	0.09^**^	0.26^**^	1											
(7) WLEIS.F3 Regulation	0.31^**^	0.06	0.17^**^	0.29^**^	0.46^**^	0.26^**^	1										
(8) WLEIS.F4 Use	0.28^**^	0.02	0.16^**^	0.25^**^	0.46^**^	0.12^**^	0.41^**^	1									
(9) WLEIS.TT	0.30^**^	0.11^**^	0.20^**^	0.32^**^	0.73^**^	0.58^**^	0.74^**^	0.76^**^	1								
(10) BDIF1 Cognitive	-0.41^**^	-0.08^**^	-0.17^**^	-0.34^**^	-0.31^**^	-0.02	-0.33^**^	-0.31^**^	-0.35^**^	1							
(11) BDI.F2 Somatic	-0.26^**^	-0.05	-0.17^**^	-0.26^**^	-0.24^**^	0.01	-0.25^**^	-0.22^**^	-0.25^**^	0.64^**^	1						
(12) BDI.TT	-0.40^**^	-0.07^*^	-0.19^**^	-0.35^**^	-0.30^**^	-0.01	-0.32^**^	-0.30^**^	-0.34^**^	0.98^**^	0.79^**^	1					
(13) SWLS.1	0.25^**^	0.01	0.18^**^	0.24^**^	0.27^**^	0.01	0.19^**^	0.22^**^	0.24^**^	-0.29^**^	-0.20^**^	-0.28^**^	1				
(14) SWLS.2	0.47^**^	0.07^*^	0.19^**^	0.39^**^	0.29^**^	0.04	0.29^**^	0.28^**^	0.32^**^	-0.43^**^	-0.30^**^	-0.42^**^	0.44^**^	1			
(15) SWLS.3	0.38^**^	0.07^*^	0.15^**^	0.32^**^	0.34^**^	0.08^**^	0.35^**^	0.30^**^	0.37^**^	-0.43^**^	-0.30^**^	-0.42^**^	0.38^**^	0.62^**^	1		
(16) SWLS.4	0.35^**^	0.04	0.22^**^	0.34^**^	0.22^**^	0.09^**^	0.26^**^	0.23^**^	0.28^**^	-0.34^**^	-0.23^**^	-0.33^**^	0.37^**^	0.54^**^	0.55^**^	1	
(17) SWLS.5	0.25^**^	0.02	0.22^**^	0.28^**^	0.17^**^	-0.01	0.16^**^	0.21^**^	0.19^**^	-0.27^**^	-0.17^**^	-0.26^**^	0.32^**^	0.41^**^	0.43^**^	0.48^**^	1
(18) SWLS.TT	0.45^**^	0.05	0.26^**^	0.42^**^	0.33^**^	0.06^*^	0.33^**^	0.32^**^	0.36^**^	-0.47^**^	-0.32^**^	-0.45^**^	0.64^**^	0.79^**^	0.78^**^	0.79^**^	0.74^**^


*MSPSS, *Multidimensional Scale of Perceived Social Support*; WLEIS, *Wong and Law Emotional Intelligence Scale*; BDI, *Beck Depression Inventory-II*; SWLS, *Satisfaction With Life Scale*; F, name of the factor; TT, total score, in the case of SWLS is each of the items; ^∗^*p* ≤ 0.05 and ^∗∗^*p* ≤ 0.01.*

### Measurement Model

Despite all the scales had adequate initial psychometric properties, due to the adaptation to the Moroccan culture, basic analyses of their properties at the item level were carried out. Mainly an analysis of discrimination (item-test correlation) and reliability of each item were performed, as well as the analysis of saturation from McDonald’s omega estimates (see Zinbarg et al., 2005). The latter did not allow assuring that all the items contributed to the measurement model, that is, to load at least to one of the factors that compounded the original factorial structure and / or to contribute to a general dimension. This analysis led us to omit items 7 (When the situation gets worse, I rely on my friends) and 12 (I can talk with my friends about my problems) of the MSPSS scale to measure social support. Also item 13 (I always know if I am happy or not) of the WLEIS scale to measure emotional intelligence and item 21 regarding to sex relations of the BDI-II scale for depression were eliminated.

In order to estimate the reliability of the scales, given the categorical format of the items, we opted for the omega index; which has also shown conceptual advantages over the alpha index (see Revelle and Zinbarg, 2008; Sijtsma, 2008). More specifically, Table 1 collects both the total, that is, the greatest lower bounds as estimates of a reliability of a test, (see omegaT column) and the hierarchical (amount of variance attributable to one common factor for all of the items, see omegaH column) variants of the omega index. However, we also provide the Cronbach alpha index, in order to facilitate comparability with other studies (see Table 1). The reliability estimates for our Moroccan sample showed values similar to those observed in the original studies (see section “Measures”). Despite some of the scales have alpha values between 0.6 and 0.7, reflecting a questionable internal consistency (Hair et al., 2014; DeVellis, 2017), these values could also be a consequence of the small number of items that compose these scales (Cortina, 1993).

Once the adequacy of all the measures was verified, it was found that the measurement model that included all the constructs enunciated as latent variables (social support, depression, emotional intelligence, and satisfaction with life) showed a good fit to the data (see Table 3).

**Table 3:** Goodness of fit indices for the measurement models.

	χ^2^ (df)	*p*	Ratio (<2)	Hoelter CN (>200)	CFI, IFI (>=0.95)	TLI, NNFI (>=0.95)	SRMR (<=0.08)	RMSEA [95% CI of RMSEA] (<=0.06)	Baseline RMSEA (>0.158)	AIC	Adj BIC	γ-Hat (>=0.95)	Adj γ-Hat (>=0.95)
MSPSS-3-Factor model	77.59 (32)	<0.001	2.42	758+	0.96+	0.95+	0.03+	0.03 [0.02–0.04]^∗^	0.1471	12510	12575	0.99+	0.99+
WLEIS-4-Factor model	304.62 (86)	<0.001	3.54	455+	0.94	0.93	0.04+	0.045 [0.04–0.05]^∗^	0.1719+	44189	44286	0.98+	0.97+
SWLS- Unidimensional	42.43 (5)	<0.001	8.49	332+	0.98+	0.96+	0.02+	0.08 [0.06–0.10]	0.3910+	20355	20384	0.99+	0.96+
BDI-II-2-Factor model	304.62 (86)	<0.001	3.54	455+	0.94	0.93	0.04+	0.045 [0.04–0.05]^∗^	0.1719+	44189	44286	0.98+	0.97+
General model	298.15 (85)	<0.001	3.51	461+	0.95+	0.95+	0.04+	0.045 [0.04–0.05]^∗^	0.1987+	64091	64158	0.98+	0.97+


*MSPSS, *Multidimensional Scale of Perceived Social Support*; WLEIS, *Wong and Law Emotional Intelligence Scale*; SWLS, *Satisfaction With Life Scale*; BDI, *Beck Depression Inventory-II*; χ^*2*^, chi square; *df*, degrees of freedom; *p*, level of signification; CN, critical N; CFI, comparative fit index; IFI, incremental fit index; TLI, tucker lewis index; NNFI, non-normed fit index; SRMR, standardized root mean square residual; RMSEA, root mean square error of approximation; CI, confidence interval; AIC, Akaike information criterion; BIC, Bayesian information criterion; γ-Hat, gamma hat index; adj γ-Hat, adjusted gamma hat index. ^∗^Indicative that the RMSEA is significant at 0.05. In all variants, “+” is indicative that the statistic meets the recommended thresholds of good fit: ρ(χ^*2*^) ≥ 0.05, χ^*2*^/df ≤ 2, [GFI CFI, IFI, NNFI] ≥ 0.95, SRMR ≤ 0.08, and RMSEA ≤ 0.06 (Hu and Bentler, 1999), [γ-Hat, adj γ-Hat] ≥ 0.95 (Fan and Sivo, 2007), Hoelter CN > 200 (Kenny, 2015). These threshold values have been included together with the indices themselves to facilitate their interpretation.*

In addition, all the parameters associated with the definition of latent variables were significant, thus confirming their statistical relevance from the indicator variables. On the other hand, when the measurement model was estimated, the freedom to freely intercorrelate all the latent variables allowed us to rule out the presence of extreme interrelations among the latent traits (the interrelation of greater magnitude was 0.754 with an average of 0.590), thus avoiding possible problems of multicollinearity (see Byrne, 2009). In the original studies on which the present study is based, all the measures that showed factorial structure exhibited non-orthogonal factors (see section “Materials and Methods”), and those of the global measurement model, mentioned above, were programmed to allow the factors to be oblique. In this way, potential problems of interpretation of the structural model due to item parceling were discarded (see Bandalos and Finney, 2001).

### Structural Model

**FIGURE 2:**
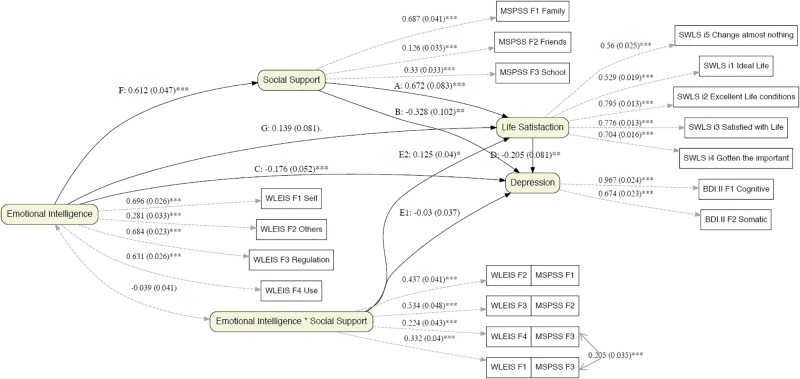
General structural model of hypothesized effects of emotional intelligence on well-being measures. The diagram includes the definition of the set of measurement (latent), structural (regressions), and Residual. Each of the paths includes the most relevant estimates: The Standardized regression weights, the standard errors (in brackets), and the signaling of those that are statistically significant (asterisks) at *p* < 0.05.

To analyze the effect of emotional intelligence on well-being measures, life satisfaction and depression, we started with a general structural model that assumes the possible modulating effect through the interaction of emotional intelligence with social support (see Figure 2). The pathways that allowed us to test our hypotheses, with direct pathways as well as two-way pathways for moderation, were then added to this model.

The chi-square difference test indicated that model specifications significantly improved model fit [χ^2^(2) = 7.32, *p* = 0.03] compared to the initial measurement model. In addition, the parameters associated with modulation (see E.1 and E.2 pathways, Figure 2) also led to significantly improved model fit [χ^2^(2) = 8.34, *p* = 0.02] compared to the reference model that lacked such parameters. This starting model (Figure 1) provided a good fit to the data (see Table 4).

**Table 4:** Goodness of fit indices for the measurements for the structural model.

	χ^2^ (df)	*p*	Ratio (<2)	Hoelter CN (>200)	CFI, IFI, TLI, NNFI (>=0.95)	SRMR (<=0.08)	RMSEA [95% CI of RMSEA] (<=0.06)	Baseline RMSEA (>0.158)	AIC	Adj BIC	γ-Hat (>=0.95)	Adj γ-Hat (>=0.95)
Starting model	328.87 (143)	0.001	2.30	667+	0.96+	0.03+	0.035 [0.03–0.04]^∗^	0.1598+	86326	86417	0.98+	0.98+
Final model	329.36 (144)	0.001	2.29	671+	0.96+	0.03+	0.035 [0.03–0.04]^∗^	0.1598+	86325	86414	0.98+	0.98+


*χ^*2*^, chi square; *df*, degrees of freedom; *p*, level of signification; CN, critical N; CFI, comparative fit index; IFI, incremental fit index; TLI, tucker lewis index; NNFI, non-normed fit index; SRMR, standardized root mean square residual; RMSEA, root mean square error of approximation; CI, confidence interval; AIC, Akaike information criterion; BIC, Bayesian information criterion; γ-Hat, gamma hat index; adj γ-Hat, adjusted gamma hat index. ^∗^Indicative that the RMSEA is significant at 0.05. In all variants, “+” is indicative that the statistic meets the recommended thresholds of good fit: ρ(χ^*2*^) ≥ 0.05, χ^*2*^/df ≤ 2, [GFI CFI, IFI, NNFI] ≥ 0.95, SRMR ≤ 0.08, and RMSEA ≤ 0.06 (Hu and Bentler, 1999), [γ-Hat, adj γ-Hat] ≥ 0.95 (Fan and Sivo, 2007), Hoelter CN > 200 (Kenny, 2015). For details see note on Table 3.*

**FIGURE 3:**
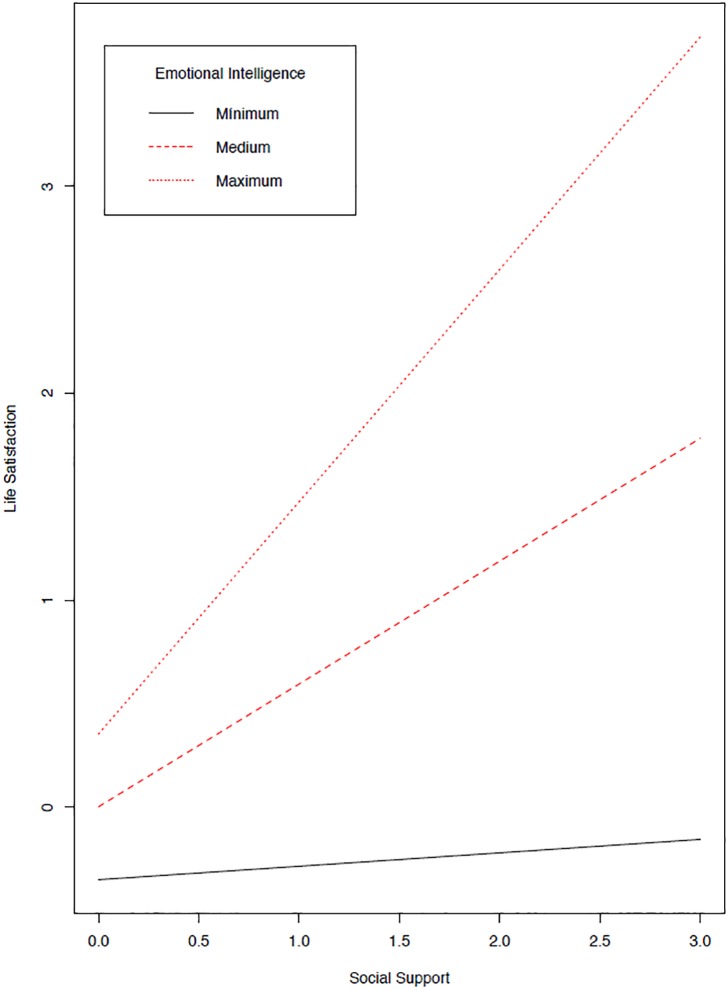
Effect of Latent interaction on Life Satisfaction. Representation of latent interactions from Products of Indicators using residual centering strategy.

The results support the core idea of the statistical relevance of the possible modulating effects of emotional intelligence with social support. However, as shown in Figure 2, only the pathway from interaction to life satisfaction was significant (E2: β = 0.125, se = 0.040, *z* = 3.120, *p* = 0.002) but not the pathway to depression (E1: β = -0.030, se = 0.037, *z* = -0.802, *p* = 0.422). See details on the interactions in Figure 3, 4. A final model identical to the original model was defined, except for the omission of the non-significant emotional intelligence and support social interaction in the prediction of the levels of depression (see Figure 5). This final model produced a good fit (see Table 4).

**FIGURE 4:**
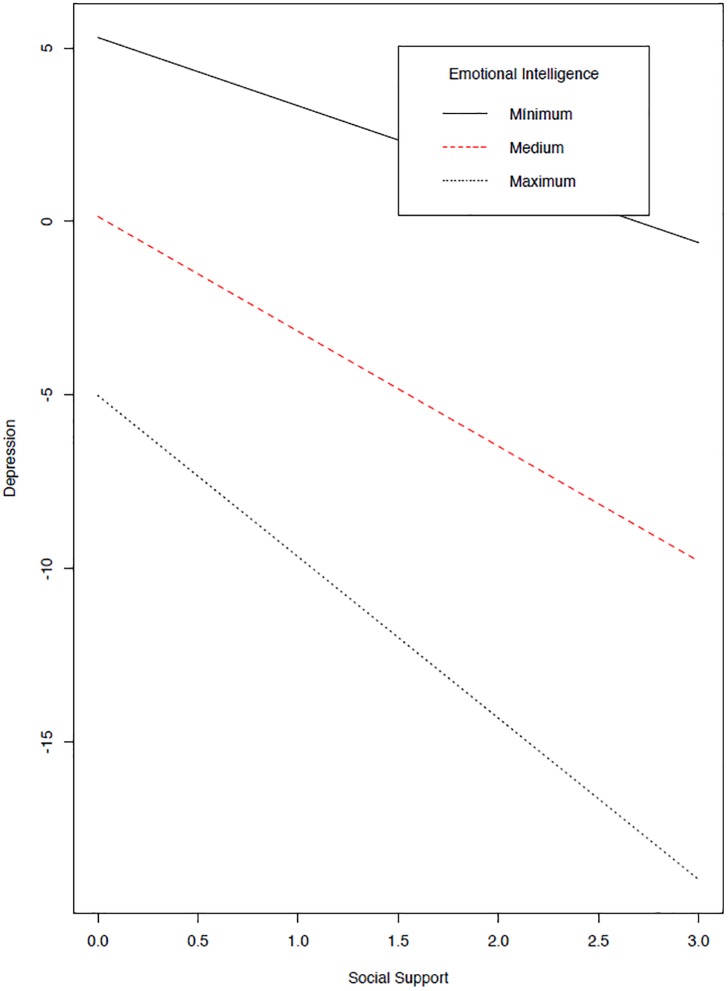
Effect of Latent interaction on Depression. As in Figure 3, the interaction corresponds to products of indicators using residual centering strategy.

**FIGURE 5:**
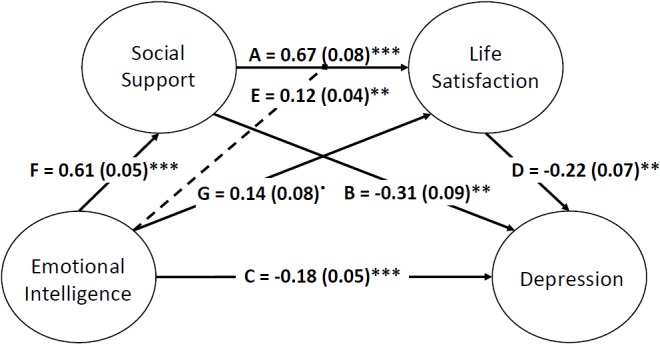
Final Model of structural relations. See notes in Figure 2.

The final model adjustment is equivalent to the starting model of the interaction; if anything, general indicators improve. The analysis based on modification confirmed that there is no misspecification in any of the parameters included in the final model.

In sum, the results show that emotional intelligence has an effect on life satisfaction, both direct (despite not strictly significant but rather approaching significance; G pathway, β = 0.144, se = 0.078, *z* = 1.847, *p* = 0.065) and indirect (interacting with social support; E pathway, β = 0.123, se = 0.039, *z* = 3.189, *p* < 0.001). See effect in Figure 3. When emotional intelligence is low, the relationship between social support and life satisfaction is practically null and in fact the slope is not statistically significant (β = 0.065, se = 0.219, *z* = 0.297, *p* = 0.767), whereas with an intermediate value in emotional intelligence, a positive relationship emerges (β = 0.594, se = 0.113, *z* = 5.232, *p* < 0.001), and maximum levels of emotional intelligence enhance this relationship (β = 1.122, se = 0.229, *z* = 4.894, *p* < 0.001). The pathways between emotional intelligence and life satisfaction have the strongest effects. The results show both the influence of emotional intelligence on social support (F pathway, Figure 5: β = 0.609, se = 0.046, *z* = 13.224, *p* < 0.001) and of social support on life satisfaction (see A pathway, Figure 5: β = 0.665, se = 0.080, *z* = 8.317, *p* < 0.001).

Moreover, emotional intelligence also has an effect on depression, but it is exclusively a direct effect (C pathway, Figure 5: β = -0.176, se = 0.050, *z* = -3.490, *p* < 0.001). As mentioned above, a modulating effect was ruled out, but a timely mediational analysis allowed us to discard a mediation effect from social support to the relation between emotional intelligence and depression (β = -0.189, se = 0.820, *z* = -0.230, *p* = 0.818). In fact, the graph in Figure 4 reveals the parallelism between the slopes for the social support depression regression at different levels of emotional intelligence. The net effect of emotional intelligence is to decrease levels of depression (see the parallel negative regression functions of Figure 4). For the prediction of depression, it also affects social support (B pathway, Figure 5: β = -0.309, se = 0.090, *z* = -3.443, *p* < 0.001) according to a moderately high value. Finally, the two well-being variables are directly related (D pathway, Figure 5: β = -0.223, se = 0.069, *z* = -3.261, *p* < 0.001). In this sense, we ruled out the possible mediated effect of the association between EI and depression through satisfaction with life, as it was not significant (β = -0.032, se = 0.138, *z* = -0.233, *p* = 0.816), and the mediation effect from social support to depression through satisfaction was discarded (β = -0.149, se = 0.586, *z* = -0.254, *p* = 0.800). That is, all types of mediation from satisfaction to depression were discarded, either from EI or from social support.

## Discussion

Due to the importance that social support has on the prediction of satisfaction with life and depression in other subsamples, the interest of testing a model of relations in Moroccan adolescents is clear. Moreover, this study contributes by adding emotional intelligence as a protector variable for positive well-being (life satisfaction) and negative well-being (depression). For the sake of clarity in the following, we revise the results according to each hypothesis.

Hypothesis 1, in which an inverse relationship between life satisfaction and depression was proposed, was supported by our results. Thus, in line with previous results in adults (Koivumaa-Honkanen et al., 2004; Boyraz et al., 2014) and adolescents (Huebner et al., 2000; Haranin et al., 2007; Lyons et al., 2014), our results show that levels of life satisfaction inversely predict levels of depression in Moroccan adolescents. The logic of this relationship could be based on the postulates of Beck’s cognitive theory of depression (Beck, 1976), which asserts that the maintenance of a series of negative cognitive self-schemas (beliefs about oneself, life and the future), together with the presence of cognitive biases (i.e., attention, memory), increases an individual’s vulnerability to depression in the future (Beck and Dozois, 2011). Thus, given that life satisfaction constitutes a global cognitive assessment (positive or negative to a greater or lesser degree) of individuals’ lives, it is logical that adolescents who maintain negative vital evaluations are those with greater depressive symptomatology and vice versa. Some authors suggest that it would be during the first years of life when these self-schemas would be established as adverse events are experienced, thus determining, along with other variables, one’s vulnerability to depression (Evans et al., 2005). This perspective would highlight the relevance of promoting adequate levels of life satisfaction to mental health in children and adolescents.

In hypotheses 2 and 3, we considered the relationship of social support with life satisfaction (positively) and depression (in a negative way). These relations are confirmed by the structural model obtained. Well-being is promoted by relations with other relevant people. This well-being implies a higher life satisfaction and a lower level of depression when social support is high. This is in agreement with previous results in other samples finding that social support increases the perceptions of well-being (Thoits, 2011) both directly and indirectly by reducing stress and other negative symptoms. Regarding depression, perceived social support protects against depression in adults (Santini et al., 2015), as well as in children and adolescents (Gariépy et al., 2016), but this study is the first to analyze this relation in a sample of Moroccan adolescents.

As for hypothesis 4, in which we predicted a positive relation between emotional intelligence and life satisfaction, the structural model confirms a significant positive relation. Our results coincide with previous works that have shown consolidated relationships between both constructs (Sánchez-Álvarez et al., 2016) and between emotional intelligence levels and the well-being of people in general (Zeidner et al., 2012). Adolescents with higher levels of emotional intelligence use adaptive strategies when dealing with uncomfortable or difficult situations (Mikolajczak et al., 2009b), which lead them to experience positive emotions when these strategies are appropriate and, similarly, to the management of negative emotions, which finally favors the preservation of a positive cognitive assessment of life in general (Palmer et al., 2002). As Schutte et al. (2002) stated, “those who are able to understand and regulate their emotions should be able to generally maintain a better outlook on life” (p. 770).

Regarding hypothesis 5, in which we predicted a negative relationship between emotional intelligence and depression, the results related to the adjustment of the model also confirm this hypothesis and therefore coincide with previous studies carried out with adolescents (Balluerka et al., 2013; Resurrección et al., 2014). Surely, the most emotionally intelligent adolescents use more effective coping strategies, which would lead them to lower levels of depression (Davis and Humphrey, 2012). In addition, they cushion the negative effect that critical vital events have on psychological maladjustment (Cha and Nock, 2009).

Our results also confirm hypothesis 6, in which a positive relationship between emotional intelligence and social support was predicted. Different studies have found similar results (Ciarrochi et al., 2001, 2002b; Balluerka et al., 2013),showing the importance of emotional intelligence in the processes of social adaptation and in the development of interpersonal relationships (Lopes et al., 2003). In fact, it has been found that both self-report and performance measures of EI contributed to the explanation of social support beyond the effects of personality (Fabio and Kenny, 2012).

Results showed a significant interaction of emotional intelligence with social support in determining levels of life satisfaction was predicted in Moroccan adolescents, thus supporting hypothesis 7. This moderating role of emotional intelligence, in interaction with different environmental variables, has already been shown in previous studies (Ciarrochi et al., 2002a; Slaski and Cartwright, 2003). Moreover, our results are in agreement with Gallagher and Vella-Brodrick (2008). They showed, in adults, that high levels of emotional intelligence contributed to the maintenance of high levels of well-being. Emotional intelligence, taking into account that social support is a buffer for stress and positively correlates with well-being (Malecki and Demaray, 2006), would contribute to the development of coping strategies that would ultimately preserve and enhance levels of life satisfaction.

The results of the adjustment of the model do not confirm an interaction of the emotional intelligence with the levels of social support in the explanation of the levels of depression in Moroccan adolescents. This implies that hypothesis 8 was not supported. Thus, alternative ways of action to those proposed by Gallagher and Vella-Brodrick (2008) should be considered. Beyond the effect that emotional intelligence can exert on levels of depression from its direct effects on levels of satisfaction with life, a mediational model in which emotional intelligence acts directly and indirectly (through its effect on levels of social support) on the levels of depression of adolescents is neither confirmed by the structural model tested in this study. This mediational model has been successfully tested in other studies (Zeidner and Matthews, 2016), confirming the key role of social support as a factor that mediates the relationships between emotional intelligence and mental health. Moreover, it distinguishes the social function that emotional intelligence has (Zeidner et al., 2016). However, the adjustment of our data to the structural model does not confirm the mediational role of social support.

In sum, our hypotheses are mainly supported. As shown in Figure 5, life satisfaction and depression, as well as social support and depression, are negatively related. Thus, the higher one’s life satisfaction and social support are, the lower his or her depression. Moreover, emotional intelligence has a direct effect on life satisfaction and interacts with social support. This finding implies that higher levels of emotional intelligence are related to a higher level of social support, and the greater the social support is, the greater the life satisfaction. Furthermore, emotional intelligence and social support separately also have direct effects on depression. Finally, social support also buffers depression, but there is no interaction effect.

These results are in agreement with those found in other samples showing that social support and emotional intelligence play a protective role in depression and the cognitive component of subjective well-being (life satisfaction). However, the analysis of their behavior in Moroccan adolescents is of great interest due to the contributions that these results have on the development of the educational Moroccan system. Education has been one of the main concerns of Moroccan authorities, given its impact on the formation of future generations and their access to an active life, along with their contribution to the growth of the country. As an example of this importance of education, the percentage of illiteracy has dropped from 54.9% in 1982 to 36.7% in 2012. Furthermore, the budget for education comprised 22.5% of the total budget in 2018, which was an increase of 9% from 2017 (Ministère de l’Economie et des Finances, 2018). These advances must be accompanied by improvements in quality. These improvements can be made in two major areas: cognitive and emotional development. The former has received a special emphasis to the detriment of the latter, which has been almost forgotten by educational practitioners. However, despite the need for the international recognition of emotional abilities in educational programs (Organisation for Economic Co-operation and Development, 2018), the lack of training of Moroccan teachers in this regard makes it impossible to implement them. Furthermore, the absence of standardized instruments in Arabic to evaluate the variables of interest makes it difficult to know how the variables relate. This study fills this gap by adapting and further analyzing several scales with Moroccan samples of adolescents and by proposing and verifying a relational model that can help researchers and teachers to more precisely clarify these relations according to their context. For example, for interventions in families and in schools, social support may be a fruitful goal, as research on the mediating role that depression can play in academic outcomes suggests important secondary benefits to depression prevention programs in school settings (Rueger et al., 2016). The same can be said about interventions that include emotional intelligence, as both variables have shown to be protective resources against depression, in addition to their beneficial effects on life satisfaction. The enhancement of protective factors, such as social support and emotional intelligence, promotes healthy youth development, thus creating healthier societies in the future.

However, despite the contribution of this study related to its analysis of a Moroccan sample of adolescents and its testing of a model of relations in which we prove that social support and emotional intelligence are protective factors of well-being, positively in increasing life satisfaction and in reducing depression, this study is not absent of limitations. The data collection process occurred much more slowly than expected. The participants were not accustomed to responding to questionnaires, and thus, they had difficulty in understanding the response procedure. To reduce the impact of this lack of knowledge, surveyors were instructed to remember the manner of response to Likert scales and the meaning of the anchors. Moreover, the neutral anchors were suppressed to reduce the acquiescence response style, as suggested by previous studies (i.e., El Rhazi et al., 2009). Another limitation is related to the absence of control of other dimensions as personality or cognitive abilities that have been related to emotional intelligence. In fact, we tried to control the influence of cognitive abilities (see Brackett and Mayer, 2003) by including the test RAVEN widely used to assess general intelligence. However, in this study, results discarded the possibility of interpreting the results observed in structural models through variation with cognitive factors.

Finally, our study is the cross-sectional design. Thus, in the future, longitudinal studies could allow for the examination of the benefits of the development of these personal variables, such as social support and emotional intelligence, to increase life satisfaction and to reduce depression in adolescents.

## Data Availability

The datasets generated for this study are available on request to the corresponding author.

## Ethics Statement

Ethical permission was obtained from the Department of Psychology and approved by the Committee at the Faculty of Letters and Human Sciences-Dhar el Mehraz of the University of Sidi Mohamed Ben Abdellah in Fez (Morocco). Once the researches received the permission to conduct the study, they applied for permission to the responsible of the Regional Academy of Education and Training, to allow the access to public schools. The administrative and education officials approved the questionnaire and procedure to be administered at public schools and gave the researches a letter to be presented at schools. In each school an internal committee composed by the school personnel informed the families and obtained their verbal consent.

## Author Contributions

EL-Z, MP-M, KE, JA-L, and BZ conceived and designed the study. BZ, KE, and SA adapted the scales to Moroccan, trained the surveyors, and collected the data. DC-D processed the data and helped with the references. MMR-Á carried out the measurements and data analyses, contributed to the conceptual model, and interpreted the data. EL-Z, MP-M, MMR-Á, JA-L, KE, OL-R, and DC-D drafted the manuscript. EL-Z integrated and coordinated the study. All authors provided substantial contributions to the work and critically revised the manuscript, approved its final version, and agreed to be accountable for all aspects of this work and its integrity.

## Conflict of Interest Statement

The authors declare that the research was conducted in the absence of any commercial or financial relationships that could be construed as a potential conflict of interest.
